# Employability as a Guiding Outcome in Veterinary Education: Findings of the VetSet2Go Project

**DOI:** 10.3389/fvets.2021.687967

**Published:** 2021-10-08

**Authors:** Martin Cake, Melinda Bell, Kate Cobb, Adele Feakes, Wendy Hamood, Kirsty Hughes, Eva King, Caroline F. Mansfield, Michelle McArthur, Susan Matthew, Liz Mossop, Susan Rhind, Daniel Schull, Sanaa Zaki

**Affiliations:** ^1^School of Veterinary Medicine, Murdoch University, Perth, WA, Australia; ^2^School of Veterinary Medicine and Science, University of Nottingham, Nottingham, United Kingdom; ^3^School of Animal and Veterinary Sciences, The University of Adelaide, Adelaide, SA, Australia; ^4^Royal (Dick) School of Veterinary Sciences, The University of Edinburgh, Edinburgh, United Kingdom; ^5^School of Veterinary Science, The University of Queensland, Gatton, QLD, Australia; ^6^School of Education, The University of Notre Dame Australia, Fremantle, WA, Australia; ^7^College of Veterinary Medicine, Washington State University, Pullman, WA, United States; ^8^Vice-Chancellor's Office, The University of Lincoln, Lincoln, United Kingdom; ^9^Sydney School of Veterinary Science, The University of Sydney, Sydney, NSW, Australia

**Keywords:** employability, veterinarian, veterinary education, competency, professional identity

## Abstract

This paper presents a mini-review of employability as a guiding outcome in veterinary education—its conceptualisation, utility, core elements and dimensions, and pedagogical approaches—through a summary of the findings of a major international project with the same aims (the VetSet2Go project). Guided by a conception of the successful veterinary professional as one capable of navigating and sustainably balancing the (sometimes competing) needs and expectations of multiple stakeholders, the project integrated multiple sources of evidence to derive an employability framework representing the dimensions and capabilities most important to veterinary professional success. This framework provides a useful complement to those based in narrower views of competency and professionalism. One notable difference is its added emphasis on broad success outcomes of satisfaction and sustainability as well as task-oriented efficacy, thus inserting “the self” as a major stakeholder and bringing attention to resilience and sustainable well-being. The framework contains 18 key capabilities consistently identified as important to employability in the veterinary context, aligned to five broad, overlapping domains: *veterinary capabilities* (task-oriented work performance), *effective relationships* (approaches to others), *professional commitment* (approaches to work and the broader professional “mission”), *psychological resources* (approaches to self), plus a central process of reflective *self-awareness and identity* formation. A summary of evidence supporting these is presented, as well as recommendations for situating, developing, and accessing these as learning outcomes within veterinary curricula. Though developed within the specific context of veterinarian transition-to-practise, this framework would be readily adaptable to other professions, particularly in other health disciplines.

## Introduction

In this paper, we provide a mini-review of the construct of employability—briefly, the ability to gain and sustain meaningful employment across the career lifespan (1, 2)—and its application in the veterinary context, particularly as a guiding outcome in veterinary education. This represents an executive summary of the findings of the VetSet2Go project, a multinational collaborative research project described in greater depth and detail elsewhere in project reports (3, 4) and related research studies (5–12). The project aim was to explore what employability means in the veterinary context, to define the capabilities most important in this context, and to create assessment tools and resources to build these capabilities (3), appropriate for use within veterinary curricula.

### Employability as a Guiding Outcome

A critical element of outcomes-based curricula is that they are designed with the end in mind, guided by specified learning outcomes (13). While in the health professions the core of this process lies in clearly articulating competencies—observable abilities of a professional integrating knowledge, skills, and attitudes (14, 15)—this itself requires clarity around *why* and *for whom* these competencies are required, i.e., the overarching outcomes, rationale, and key stakeholders guiding curriculum design. Where elaborated for published veterinary competency frameworks, these drivers have tended to focus primarily on graduate preparedness to ensure patient safety and meeting societal expectations. For example, the stated intention of the Competency-Based Veterinary Education (CBVE) framework is to “prepare graduates for professional careers by confirming their ability to meet the needs of animals and the expectations of society” [(16), p. 1], while also incorporating stakeholder expectations of workplace performance, including preparing graduates for the “complex roles of today's healthcare professionals” [(17), p. 580]. Similarly, the Royal College of Veterinary Surgeons' *Day One Competences* document (18) describes “the knowledge, skills and attributes required of veterinary students upon graduation to ensure that they are prepared for their first role in the profession and safe to practise independently” [(18) p. 3]. These frameworks are thus primarily focused on quality assurance to protect patients and clients, and only secondarily on other benefits of preparedness such as employer or graduate satisfaction.

Several competency frameworks have outlined a broader set of outcomes and rationales. Bok et al. (19) used a Delphi procedure to develop an integrative framework that intentionally reduced the prominence of technical expertise, listing this as only one domain alongside communication, collaboration, entrepreneurship, scholarship, public health and welfare responsibilities, and personal development. The drivers cited for this broader curriculum framework were: societal changes placing increasing importance on “generic” or professional competencies, closing the transitional gap due to inadequate or mismatched preparation for work, and to “future-proof” learning beyond graduation (19). Similarly, the 2011 NAVMEC report (20) outlined a framework weighted towards professional (non-technical) competencies, in response to evolving societal needs and challenges including technology, financial sustainability, and lifestyle balance. The report framed veterinary education as increasing value, not only in the general sense of developing skills valuable to society and to employers, but also which for graduates personally “increases their value in the veterinary medical market” [(20) p. 31]. These broader frameworks thus address the needs of multiple stakeholders including the graduate themselves, such as smooth transition to work, broad career opportunities, well-being, and sustained financial and professional success.

Arguably, this broader set of outcomes aligns more fully with the construct of *employability* than with competency, particularly when the latter is more narrowly defined around observable abilities (14). Bell et al. (6) outlined the case for embracing employability as an overarching goal of veterinary education, as a complement to the essential aims of competency and professionalism. As an educational “lens,” employability brings particular focus on transition to work, career success and satisfaction, long-term sustainability, well-being and resilience, and human potential. This focus aligns with multiple contemporary challenges for veterinary education and for the profession, including: evolving views of both the nature of employment and the “work-readiness” role of higher education, driving the so-called “employability agenda” (21); concerns about the future sustainability of the veterinary workforce and the profession, as highlighted in various industry reports in the US and UK (20, 22–25); and the need to support well-being in the face of elevated mental health risks (26–29).

## Defining and Conceptualising Employability

While multiple definitions of employability exist, none have emerged as dominant (30). Most definitions reference the capacity to gain and sustain employment (2, 31–33), through possession of a set of assets seen as desirable to potential employers (2, 34, 35). Broader and more holistic definitions are focused less on employment outcomes and employer-led “key skills” and more towards contextual person- and process-centred perspectives (36, 37). The VetSet2Go project adapted the widely-cited definitions of Knight and Yorke (38) and Dacre Pool and Sewell (34) to derive a working definition of employability in the veterinary context as: *a set of adaptive personal and professional capabilities that enable a veterinarian to gain and sustain employment, contribute meaningfully to the profession and develop a professional pathway that achieves satisfaction and success* (6). It is particularly this framing around personal outcomes of satisfaction (34) and meaningfulness (31, 39, 40) in work, and the longer-term trajectory of adaptive, sustainable development that distinguishes employability from “day-one” competency and professionalism. Recognising the reciprocity of these employer- and employee-led perspectives, as well as the complexity of the multiple roles and stakeholder needs a veterinary professional must fulfil (41), an alternative definition developed from the project was: “*an individual's capacity to sustainably satisfy the optimal balance of all stakeholder demands and expectations in a work context, including their own”* (12).

### Elements of Employability

Recent reviews unpacking the conceptual complexity of employability have been published by Williams et al. (30) and Small (21). Widely used conceptual models of employability include Knight and Yorke's (35) USEM model and Dacre Pool and Sewell's CareerEDGE model (34). Some authors subdivide employability assets into various forms of *capital*, describing properties of an individual that elicit employment demand or provide added functionality to an employer (30, 42, 43). These include human capital (knowledge, skills and training), social capital (connexions and networks), cultural capital (experiences enhancing cultural fit), and psychological capital (psychological strengths) (30). Major psychological factors include adaptability or flexibility (32, 44) and willingness or work ethic (33). Some recognise emotional intelligence (34) or interpersonal skills (21, 31) as a distinct element of employability capital spanning across learned skills and psychological traits. These psychological factors, respectively, align loosely to the major personality dimensions of *openness, conscientiousness*, and *agreeableness* as defined in the Five-Factor Model of personality (45), which have all been positively associated with work performance (46).

These various forms of capital are translated into employability outcomes by a further process dimension of *career development*, representing the process of navigating oneself into future roles (30). This includes a number of elements including *signalling* (ability to articulate and present assets) and *self-management* (self-awareness of goals and values). The core developmental processes in employability include reflective self-awareness, which in turn builds self-beliefs (self-efficacy, self-esteem, self-confidence) (34, 35, 47). Rust and Froud (47) argued for critical self-awareness or *personal literacy* as a universal meta-attribute or “master key” vital to both employability and academic learning. These central processes are notably similar to those in professional identity formation; from this perspective employability is mainly an identity project (37, 48–50).

## A Veterinary Employability Framework

A major aim of the VetSet2Go project was to develop an employability framework for veterinary education, defining the capabilities most important to employability in this context. The framework was informed by evidence from multiple stakeholder perspectives including a best-evidence systematic review (51); interviews and focus groups of employers (5, 10), recent graduates (5), and clients (9); large international surveys of clients (9) and other stakeholders (veterinary employers, employees, colleagues, academics, industry bodies) (11); and an aligned subproject exploring veterinary career motivations, resilience, and well-being (7, 8, 52). These various strands of stakeholder evidence were integrated through a consensus process involving the project team and an expert Delphi procedure (3).

**Figure 1:**
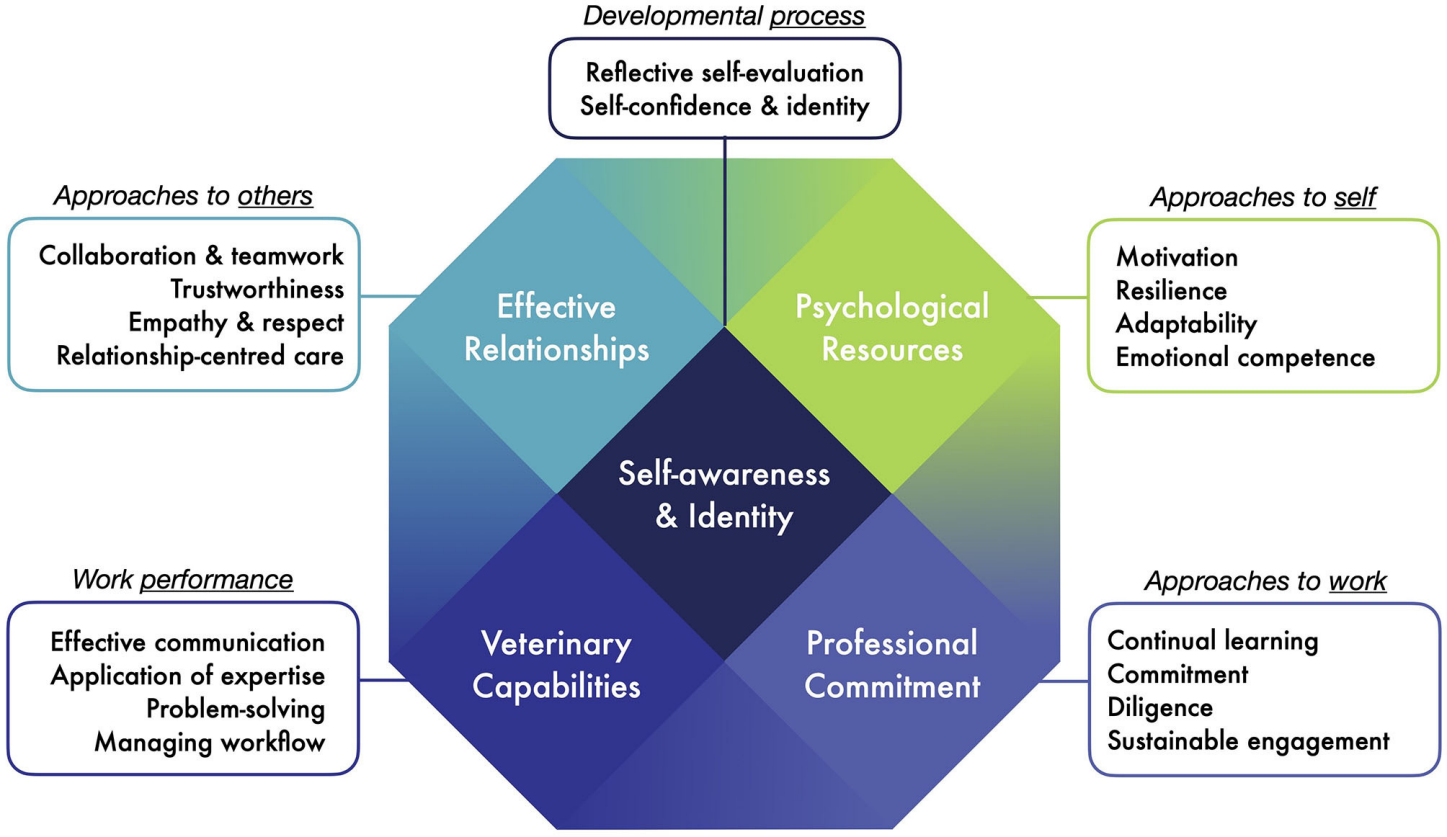
The “crystal” model of veterinary employability, with five overlapping domains and 18 aligned capabilities.

The derived framework contains 18 key capabilities consistently identified as important to employability in the veterinary context (Table 1). The term *capability* (71) is used to distinguish these from competencies or “skills,” and to signal their enabling, potential, and contextual nature. These are aligned to broad, overlapping domains, or dimensions: *veterinary capabilities* (task-oriented work performance), *effective relationships* (approaches to others), *professional commitment* (approaches to work and the broader professional “mission”), *psychological resources* (approaches to self). These are activated by a central process of reflective *self-awareness and identity* formation. These domains overlap somewhat to form an “employability crystal” model (Figure 1). For example, communication is both a “hard” discipline-specific clinical skill and a “soft” relational skill. The alignment of these domains to elements from the broader employability literature such as human and psychological capital (30), willingness (33), metacognition, and efficacy beliefs (34, 35) are indicated in Table 1. The conceptual basis for the five dimensions of the crystal is outlined by Cake et al. (12). These dimensions reflect a balance between recognising employability as established partly from a work (task-oriented) context, and partly from a human (psychological/interpersonal) context; partly from shorter-term efficacy and partly from longer-term sustainability (71); partly from possession of a set of “assets” or capital, and partly from a process of growth and identity formation (37, 49, 50). As in Harden and others' three circle model of medical education outcomes (13), employability is conceived as based partly in work performance, partly in *approaches* to performance (broadly grouped as approaches to work, to others, and to self), and partly in overarching “meta-competencies” (72) including reflective self-awareness.

**Table 1:** The 18 capabilities and five domains identified as consistently important to employability in the veterinary context, with exemplar descriptions, key published evidence from the veterinary literature, and aligned elements from the broader employability literature.

**DOMAIN, Capability**	**A veterinarian who:**	**Key evidence in veterinary context**	**Aligned elements from employability literature**
**EFFECTIVE RELATIONSHIPS**			
Collaboration and teamwork	Fits into and supports an effective veterinary team; works with others collaboratively towards shared goals; is friendly and personable	Stakeholder surveys (11, 51) and consensus (53), Delphi process (3), employer selection (10, 54), employer satisfaction (55), transition to practise (5)	Interpersonal qualities (21); interacts with others (31); social/interpersonal compatibility (rewarding to deal with) (33); emotional intelligence (34); values (49); agreeableness
Trustworthiness	Builds trust through honesty, transparency, integrity	Stakeholder surveys (11, 51, 56) and consensus (53), Delphi process (3), client satisfaction (9)	
Empathy and respect	Is attentive to others' feelings, perspectives and concerns; is non-judgmental, respects diversity of opinion, and worldview	Stakeholder surveys (11, 51) and consensus (53), Delphi process (3), client satisfaction (22, 57–59), veterinarian satisfaction (60)	
Relationship-centred care	Bases healthcare approaches in human relationships and decision-making in partnership; respects the human-animal bond	Stakeholder surveys (11, 51) and consensus (53), client satisfaction (9, 57, 61), client adherence (61)	
**VETERINARY CAPABILITIES**			
Effective communication	Is a clear and effective communicator (verbal, non-verbal, written); listens and seeks understanding; confidently discusses difficult issues including financial aspects of care	Stakeholder surveys (11, 51) and consensus (53), Delphi process (3), employer selection (10, 54), employer satisfaction (55), client satisfaction (9, 57–59, 62), client adherence (61, 63), veterinarian satisfaction (60), transition to practise (5, 64)	Human capital (30, 43); understandings and skilful practises (35); discipline-specific skills (31, 34, 65); generic skills (34, 65); occupational expertise (44); capability (32); able to do the job (33); performance (49)
Application of expertise	Inspires confidence through compassionate animal handling, sound practical skills, and application of specialised knowledge	Delphi process (3), transition to practise (5, 64), employer selection (10), client satisfaction (9)	
Problem-solving	Evaluates evidence in support of clinical reasoning and problem-solving; can make decisions despite incomplete information; uses good judgment and “common sense”	Stakeholder surveys (51, 64) and consensus (53), Delphi process (3), employer satisfaction (22, 55), employer selection (10), client satisfaction (9)	
Managing workflow	Is self-organised in their work; manages priorities and uses time efficiently and productively; uses initiative; is independent	Stakeholder surveys (51), Delphi process (3), employer selection (10), transition to practise (66)	
**PROFESSIONAL COMMITMENT**			
Continual learning	Is keen to learn, open to feedback, and strives for improvement and best practise	Stakeholder surveys (11, 51) and consensus (53), Delphi process (3), transition to practise (5), employer selection (10, 54, 56), client satisfaction (9)	Professional development (67); develop self (31); propensity to learn (32)
Commitment	Is committed to the veterinary mission, including quality care and welfare, and to organisational goals; takes responsibility	Employer selection (10), client satisfaction (9), Delphi process (3)	Willingness (33, 68); work ethic (33); professional maturity (68); personal investment (42); conscientiousness (33)
Diligence	Is hard-working, persistent, reliable; gives attention to detail and quality assurance	Stakeholder surveys (11) and consensus (53), Delphi process (3), transition to practise (5), employer selection (10)	
Sustainable engagement	Sustains an energetic connexion with their work; balances and refreshes their interest, passion and enthusiasm for work with other needs; is self-sustaining	Stakeholder surveys (11, 51), Delphi process (3), transition to practise (5)	Balance (44)
**PSYCHOLOGICAL RESOURCES**			Psychological capital (30, 42)
Motivation	Finds motivation and purpose in their work; is self-motivated and intrinsically driven	Stakeholder surveys (11)	Ambition, drive (33); meaningfulness (1)
Resilience	Deals with pressure and adversity; draws on personal and contextual resources, and utilises strategies to navigate challenges and sustain well-being	Stakeholder surveys (11, 51) and consensus (53), Delphi process (3), transition to practise (5, 64), employer selection (10)	Resilience (30, 32, 42, 48)
Adaptability	Is flexible in dealing with change, uncertainty, and shifting priorities; is open-minded	Stakeholder surveys (51) and consensus (53), Delphi process (3)	Adaptability (32, 42, 43, 67); personal flexibility (43, 44); openness (32, 33)
Emotional competence	Is able to navigate emotional situations and self-regulate emotional responses; remains calm	Stakeholder surveys (11, 51), Delphi process (3), transition to practise (5), client satisfaction (9)	Emotional intelligence (34); socio-relational competencies (67)
**SELF-AWARENESS AND IDENTITY**			
Reflective self-evaluation	Is aware of their own strengths and limitations, reflective and learns from experience; is self-aware of emotional responses and behaviours	Stakeholder surveys (11, 51), Delphi process (3), work engagement (69, 70), employer selection (10, 56), client satisfaction (9), transition to practise (64)	Metacognition (35); reflection and evaluation (34); develop self (31); personal literacy (47); self-management skills (43, 65)
Self-confidence and identity	Has positive self-esteem and self-belief, anchored in a professional self-concept based on personal values, beliefs, and goals	Stakeholder surveys (51) and consensus (53), income (23), veterinarian satisfaction (60), work engagement (69, 70), transition to practise (5), employer selection (10)	Self-esteem, self-confidence, self-efficacy (34); efficacy beliefs (35); perceived employability (43); identity (32, 37, 48–50); identity capital (42)

A number of important caveats apply to this employability framework. While it includes elements of competency and professionalism, it is intended to complement and not replace more comprehensive frameworks for these essential outcomes (16, 18, 19). It is primarily oriented to graduate-level clinical veterinary practise, so omits some competencies known to be important in mid- to late-career including business skills (5, 73) or in other veterinary work contexts (e.g., research skills). It also omits some process elements known to be important for employability more broadly though not prominent in the veterinary context, including career management (“navigating the world of work”) (31, 65), signal management (e.g., job applications, interviews) (42, 43), and social capital (e.g., networking) (30, 43).

## Pedagogical Approaches

Embracing employability as a core guiding outcome offers both opportunities and challenges for veterinary education. Structural barriers in veterinary curricula may potentially limit student and faculty engagement with employability. The strongly vocational and heavily accredited nature of healthcare degree programs, combined with high graduate employment rates, may encourage the false assumption that employability development occurs automatically. Over-full and content-heavy curricula may leave employability easily crowded out by more traditional disciplinary outcomes (74). Faculty may resist giving up curriculum space to content they view as “soft,” or may lack the capacity to confidently teach it (48). Another potential pitfall is mismatch with the hidden curriculum, if staff role models do not “walk the talk.”

Overcoming these barriers requires a clearly articulated and locally relevant definition, rationale, and conceptual framework for employability, as well as professional development for faculty to enable collective ownership and a shared approach (36). As in the VetSet2Go project, it is recommended to adopt a broad, holistic definition of employability (i.e., beyond just employer-led “key skills”), and a guiding rationale that stretches beyond initial employment and “work-readiness” to include success, satisfaction, meaning, sustainability, and balance. Since employability pedagogy requires “slow” learning approaches best integrated across multiple reflection cycles, whole-of-course, embedded, and vertically integrated strategies are more likely to succeed than stand-alone or “bolt-on” approaches (36). Employability should be revisited at multiple points across the program, to gain the benefits of both early awareness, and experiential learning in authentic clinical contexts, which may alter student confidence (75). Employability learning may be delivered in a variety of modes including workshops and group discussions, team-based tasks, reflective writing and portfolios, role plays and simulations, mentoring programs, “story-telling” in guest seminars, clinical rotations, work-integrated learning placements, and extracurricular programs.

### Learning Outcomes

The need to articulate guiding learning outcomes for employability without reducing their complexity is recognised as a major challenge for employability pedagogy (48). Similarly, the need to tolerate the complex, overlapping, and “fuzzy” outcomes typical of employability may challenge competency-based systems. Only the minority of employability capabilities are “competencies” in the narrow sense of observable abilities; the majority are psychological or attitudinal factors (e.g., self-beliefs, habits, attitudes, values, metacognition) thus may defy precise rubrics or measurement.

Despite this complexity and ambiguity, it remains important to make employability learning outcomes explicit in curricula, ideally within program-level learning outcomes. While employability outcomes overlap considerably with those based in competency and professionalism, these overlapping constructs are better treated as distinct “lenses” to explore all the dimensions of a successful veterinary professional rather than wedging employability into existing competency or professionalism frameworks (6). While accountability- or altruistic service-based framings of professionalism may seem counter to some aspects of employability, employability is compatible with professionalism framed as professional identity formation in the sense of “becoming” a professional (76); these share a similar core process and pedagogy (41, 77).

### Centre on Self

Employability pedagogy should be centred on self-awareness, reflective self-evaluation, and identity formation. These processes form a “master key” to simultaneous development of employability, competency, and professionalism. While awareness of *limitations* is emphasised in the latter frameworks, in employability self-awareness equally builds awareness of *strengths* to be harnessed or “activated,” as well as vulnerabilities to target for further development. Self-awareness also builds the ability to articulate or present assets to potential employers (e.g., in a *curriculum vitae*, portfolio, or interview).

The idea of “finding fit” is highlighted in the employability literature (30, 48), aligning with Viner's (78) premise that long-term success in the veterinary profession requires congruence between professional objectives and personal values. In contrast to normative frameworks such as competency, viewing employability as finding fit highlights the uniqueness of students' capability sets, personality traits, and core values and beliefs. This personalised and contextual view of employability removes normative thresholds (38), such that no-one need be judged “unemployable” but rather yet to find best fit with a professional niche and culture that mutually values them and that they also value (71).

### Assessment

Assessment of employability is recognised as challenging, partly because of the predominance of summative, criterion-driven approaches to assessment (38). Some of the more ability- or behaviour-based aspects of employability may be suited to summative assessment, such as direct observation or longitudinal evaluations in authentic workplace contexts, though these may be limited by low reliability (79). Other aspects of employability based more in attitudes, values, or metacognition may be better targeted formatively through guided reflection, experiential learning, mentoring, and rich feedback. Suitable assessment methods include reflective journals, portfolios, self-assessment rubrics, direct observation in a workplace, and supervisor and peer feedback (38, 79). For these more personal aspects, there may be no threshold expectation of capability, but rather only the expectation that each student has developed an appropriate level of self-awareness.

The VetSet2Go project concluded that the most feasible and fruitful approach to assessment for employability in veterinary education is likely to be multiple cycles of guided self-reflection complemented by rich, multisource feedback. A free online self-assessment tool for veterinary employability and associated resources were developed for the project^1^. An example of the implementation of this tool and its face validity was provided by Stalin (75). Since the ability to self-assess has inherent limitations, rich multisource feedback plays an important role in calibrating and triangulating self-evaluation, given that others' perceptions necessarily form a key part of employability as a process of social validation. Use of multiple “low-stakes” assessments from different perspectives (i.e., multisource feedback) has been shown in other contexts such as professionalism to overcome the error and bias in subjective assessments (80). The extensive requirement for clinical experience in veterinary degree programs, extended by mandatory extramural placements (i.e., work-integrated learning, WIL) in some countries, creates valuable opportunities for authentic employability learning if paired with an efficient method for gathering rich feedback from supervisors and observers. In this sense employability offers a solid shared framework for engagement of external partners and mentors in veterinary education. Ideally, employability outcomes should be addressed in programmatic outcome evaluation and graduate feedback.

## Discussion

This mini-review highlights the role and utility of employability as a broad and holistic guiding outcome in veterinary education, as a complement to the narrower paradigms of competency and professionalism. Major differences between employability and the latter outcomes include employability's broader focus inclusive of diverse career paths (via “transferable” skills) and multiple definitions of success. Another difference is its balance across the needs of multiple stakeholders, most notably the learner/graduate themselves, thus inserting “the self” as a major stakeholder and bringing attention to resilience and sustainable well-being. The additional focus on personal outcomes of success, satisfaction, meaningfulness, and sustainability in future employment (31, 34, 35, 71) balances existing frameworks that focus primarily on quality assurance and task-oriented efficacy (“work-readiness”) at the point of graduation. Another distinction is employability's greater emphasis on awareness of strengths as well as limitations, and on exploring and finding “fit.”

The breadth and complexity (multidimensionality) of employability offers both benefits and challenges in veterinary education. Employability's focus on implicit attitudes and “approaches” more so than readily measured abilities may require extra attention to formative, subjective assessment methods such as self-reflection and multisource feedback. Another challenge lies in defining explicit outcomes for employability development without reducing these to a list of “key skills.” The five domain conceptual model developed for the VetSet2Go project provides one possible solution, in particular its central focus on the process element of reflective self-awareness and identity formation. This may be more compatible with professionalism development based more in professional identity formation than in virtue- or behaviour-based models (76, 81).

The recommendations of the VetSet2Go project (3, 6) were to frame employability in veterinary education as focused on success and satisfaction in meaningful employment, more than just “getting a job,” and on sustainability as well as efficacy. Employability depends more on attitudes and “approaches” more than key skills, and on a central self-awareness and growth *process* as well as possessed “assets” (50). It requires personalising of professional learning and “finding fit,” and *balancing* the perspectives of multiple stakeholders including employers, clients, colleagues, and particularly (centrally) the employee/graduate themselves. Focusing on these aspects through employability, as a complement to the existing frameworks of competency and professionalism, offers multiple potential benefits in veterinary education including smoother transition-to-practise; sustainable career satisfaction; well-being, resilience, and life balance; broadening and diversification of career opportunities; and overall graduate success.

## Author Contributions

MC and MB drafted the manuscript. All authors, as members of the VetSet2Go project team, contributed important intellectual content within the research, perspectives, critical analysis and conceptual framework presented, contributed to manuscript revision, read, and approved the submitted version.

## Funding

The VetSet2Go project was supported by the Australian Government, Office for Learning and Teaching, Grant Number ID15-4930.

## Conflict of Interest

The authors declare that the research was conducted in the absence of any commercial or financial relationships that could be construed as a potential conflict of interest.

## Publisher's Note

All claims expressed in this article are solely those of the authors and do not necessarily represent those of their affiliated organizations, or those of the publisher, the editors and the reviewers. Any product that may be evaluated in this article, or claim that may be made by its manufacturer, is not guaranteed or endorsed by the publisher.

## Footnotes

^1^https://www.vetset2go.edu.au/for-learners.
